# Study on the genetic damage caused by cadmium sulfide quantum dots in human lymphocytes

**DOI:** 10.1515/biol-2022-0054

**Published:** 2022-05-11

**Authors:** Haiping Liu, Huajie Liu, Haiyan Liu, Duo Zhang, Qian Wang, Shuang Li, Yanhua Cao, Qingzhao Li

**Affiliations:** School of Public Health, North China University of Science and Technology, No. 21 Bohai Road, Caofeidian, Tangshan, Hebei, China; School of Chemical Science and Engineering, Tongji University, No. 1239 Siping Road, Shanghai 200092, China; Scientific Research Department, North China University of Science and Technology, No. 21 Bohai Road, Caofeidian, Tangshan, Hebei, China; School of Clinical Medicine, North China University of Science and Technology, No. 21 Bohai Road, Caofeidian, Tangshan, Hebei, China; School of Stomatology, North China University of Science and Technology, No. 21 Bohai Road, Caofeidian, Tangshan, Hebei, China

**Keywords:** cadmium sulfide quantum dots, DNA damage, comet test, micronucleus test, chromosome aberration test

## Abstract

Cadmium sulfide quantum dots (CdS QDs) are being developed for sensors, fluorescent probes, and other platforms and are attracting increasing attention. Given the growing demand for QDs, it is clear that there is a need to understand their potential toxicity to organisms. However, little is known regarding the genotoxicity of CdS QDs to humans. Therefore, this study used CdS QDs as the research object, cultured human peripheral blood lymphocytes, and randomly divided them into a control group, CdS I group (CdS QDs), and CdS II group (CdS QDs coated with thioglycolic acid). After cultivation, we measured the olive tail distance, tail length, tail DNA%, lymphocyte micronucleus rate, and aneuploid rate. The comet test results indicated that the indices of the QD group were significantly larger than those of the control group (*P* < 0.05). The results of the micronucleus and chromosome aberration tests showed that the lymphocyte micronucleus rate and chromosome aneuploid rate in the QD group were significantly increased (*P* < 0.05) compared with those in the control group. In conclusion, CdS QDs have certain genotoxicity to human peripheral blood lymphocytes, and the DNA damage caused by CdS QDs encapsulated with thioglycolic acid is less severe than that caused by nonencapsulated CdS QDs.

## 1 Introduction

Nanoparticles (NPs) generally refer to particles with at least one dimension, sized between 1 and 100 nm. They have some novel properties, such as a small size effect, surface interface effect, and quantum size effect, which enable their use in unique applications. Many artificially synthesized NPs have become important tools for scientific research. Among them, the application of semiconductor nanocrystals, called quantum dots (QDs), has attracted increasing attention [1]. QDs are mainly used in solar cells, optoelectronic devices, chemical catalysis, fluorescent probes, and sensors [2,3,4], and they have potential diagnostic and therapeutic effects in the field of biomedicine [5,6,7]. For example, QDs coupled with antibodies have been used to distinguish normal cells from cancer cells [8]. However, the potential threat of QDs to human health hinders their widespread application in life science [9].

Studies have shown that QDs are released into the environment as waste from QD synthesis and leakage during processing and transportation. Additionally, QDs in single electronic devices and various optoelectronic devices can be released into the environment through device use or waste treatment. QDs in the air have a strong adsorption capacity and easily adsorb gas or other particles and react with them. There are three main ways for QDs to enter the human body: the respiratory tract, digestive tract, and skin [10,11,12]. QDs entering the body can avoid phagocytosis of the immune system and accumulate in some target organs. They can also cross different biological barriers and be transported to other tissues and organs of the body, resulting in systematic health effects. Therefore, it is necessary to consider the negative effects of nanomaterials while paying attention to their beneficial biological effects.

Studies have shown that cadmium sulfide quantum dots (CdS QDs) have potential toxicity to organisms, can be distributed in various systems in organisms, and mainly gather in the lung and spleen [13,14,15]. The toxicity mechanism of CdS QDs is mainly related to the induction of reactive oxygen species (ROS) and other free radicals; the inhibition of antioxidant enzyme activity; the imbalance of oxidation and antioxidant systems; and the destruction of the integrity of proteins, nucleic acids, and membranes [16]. However, there are few studies on the genotoxicity of CdS QDs to humans. Therefore, it is of great significance to study the genotoxicity of CdS QDs in humans. Studies have shown that coating the surface of QDs can protect the core of QDs, increase the stability of QDs, and slow their toxic effects on the body, which may remove obstacles to the application of QDs in various fields. CdS QDs are hydrophobic materials and are not easily excreted in the urine. Thioglycolic acid is water-soluble and can be directly excreted in urine through kidney metabolism. Therefore, we chose thioglycolic acid to wrap CdS QDs, which can increase the stability of QDs and reduce their accumulation in various organs to reduce their toxicity.

This study investigated the genotoxicity of CdS QDs in human peripheral blood lymphocytes. The DNA damage caused by CdS QDs and thioglycolic acid-encapsulated cadmium sulfide QDs in human peripheral blood lymphocytes was analyzed through comet, micronucleus, and chromosome aberration tests to provide laboratory data and a scientific basis for the study of the reproductive and genetic toxicity of CdS QDs.

## 2 Materials and methods

### 2.1 Main instruments and reagents

HEPA carbon dioxide incubator steri-cycle CO_2_ (United States THERMO), a JA2003 electronic balance (Shanghai Jingke), a BX-41 fluorescent camera system (Japan OLYMPUS), steady flow programmable electrophoresis (Beijing Liuyi Instrument Factory), a DK-8D digital display constant-temperature water bath (Jintan Medical Instrument Factory), and a KDC-1044 low-speed centrifuge (Keida Innovation Co., Ltd., Zhongjia Branch) were used.

Low-melting-point agarose (SIGMA), normal-melting-point agarose (SIGMA), heparin, ethidium bromide, RPMI 1640 (GIBCOL), 1640 medium (GIBCOL), fetal bovine serum (GIBCOL), heparin, and plant blood coagulation prime were used.

### 2.2 Synthesis of CdS QDs

**Figure 1:**
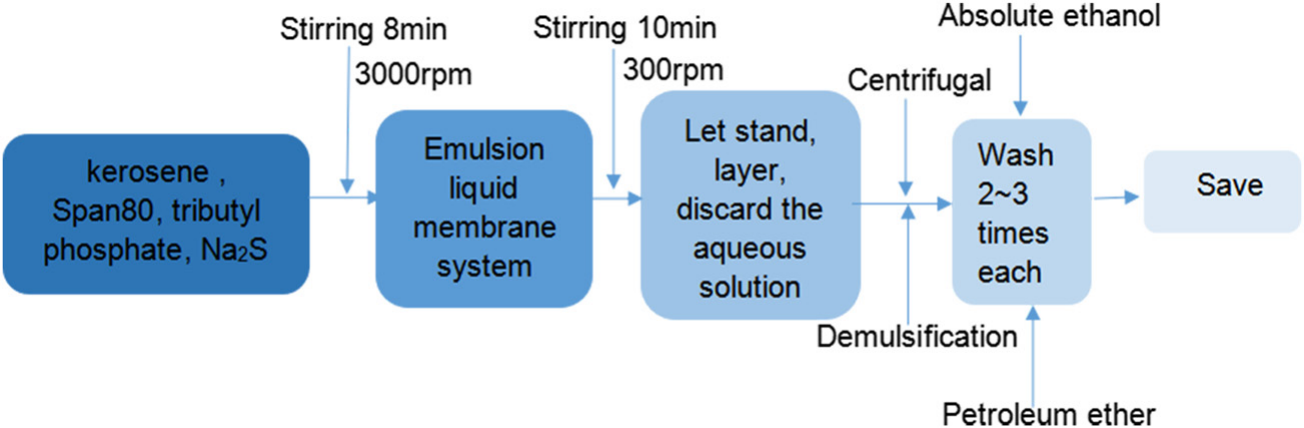
Synthesis of CdS QDs.

CdS QDs were synthesized by the emulsion liquid membrane method (Figure 1). The method is simple and easy to implement, does not require high temperature or high pressure, and can control the monomer particle size. At the same time, the raw material has low toxicity and is relatively stable at room temperature. Thus, it is an efficient “green” synthesis method. The specific method of synthesis was as follows: 50 mL of (0.2 mol/L) Na_2_S solution, 40 mL of kerosene, 5 mL of surfactant Span80 and 5 mL of tributyl phosphate were mixed in a beaker and stirred at 3,000 rpm for 8 min to form a stable emulsion liquid membrane system. Then, 50 mL of the prepared emulsion liquid membrane was added to 100 mL (0.1 mol/L) of CdCl_2_ and stirred at 300 rpm for 10 min, and stirring was then stopped. After delamination, the aqueous solution was discarded, and the remaining emulsion was centrifuged. The emulsified liquid membrane was demulsified by a centrifuge, the precipitate was washed 2–3 times with petroleum ether and absolute ethanol, and the precipitate was transferred to a bottle. The particle size of the CdS QD monomer obtained by the emulsion liquid membrane method was 6–8 nm, and the shape was granular. CdS QDs encapsulated by thioglycolic acid were provided by the School of Environmental Science and Engineering, Nankai University. The particle size of the monomer was 6–8 nm, and the shape was granular. Analytical Na_2_S and CdCl_2_ were purchased from Beijing Hengye Zhongyuan Chemical Co., Ltd. (Beijing, China); tributyl phosphate was purchased from Tianjin Damao Chemical Reagent Factory (Tianjin, China); Span80 was purchased from Jiangsu Nantong Chenrun Chemical Co., Ltd.; petroleum ether was purchased from Tiande Fine Chemical Co., Ltd. (Zibo, China); and anhydrous ethyl alcohol was purchased from Tianjin Windboat Chemical Technology Co., Ltd. (Tianjin, China).

### 2.3 Preparation of CdS QD suspension

CdS QDs and CdS QD powder coated with thioglycolic acid were autoclaved and then prepared into a suspension in 1640 medium containing 20% fetal bovine serum. After 48 h of ultrasonic vibration dissolution, the mother liquor with uniform dispersion was obtained. Then, it was stored at 4°C until used.

### 2.4 Cell culture and processing

Twenty milliliters of venous blood from a healthy adult male (without a smoking history and who signed an informed consent form) were obtained. A 0.4 mL blood sample was added to 4.6 mL of RPMI 1640 (containing 20% calf serum, 100 U/mL penicillin, and 2% PHA) culture medium and then randomly divided into a control group, CdS I group (CdS QD group), and CdS II group (CdS QD group wrapped in thioglycolic acid). The CdS I group was supplemented with uniformly dispersed CdS QD mother liquor, and the CdS II group was supplemented with uniformly dispersed thioglycolic acid-coated CdS QD mother liquor. The final concentration of the sample suspension of the two groups was 2 µg/mL, the control group was treated with the same volume of normal saline, and the three groups were cultured in a 37°C, 5% CO_2_ incubator.


**Informed consent:** Informed consent has been obtained from all individuals included in this study.
**Ethical approval:** The research related to human use has been complied with all the relevant national regulations, institutional policies, and in accordance with the tenets of the Helsinki Declaration, and was approved by the ethics committee of North China University of Science and Technology (Ethics approval number: 2021024; Ethics approval date: 20210412).

### 2.5 Experimental methods

#### 2.5.1 Comet test

In this study, a comet test was used to assess the extent of DNA damage caused by CdS QDs [17]. The principle is that the damaged DNA breaks and its superhelix structure is damaged because the molecular weight of the DNA fragment is very small; it can leave nuclear DNA in the electrophoretic field and move to the anode in the gel molecular sieve, forming a comet image. The more seriously the DNA is damaged, the more broken strands and variable fragments will be produced, and the smaller the broken strands will be. Under the same electrophoresis conditions, more DNA will migrate to the tail, and the longer the migration distance will be, which manifests as an increase in tail DNA% and tail length. Olive tail distance is a composite index of tail DNA% and migration distance, which can accurately and sensitively respond to DNA damage. Therefore, the severity of DNA damage caused by QDs can be comprehensively judged by three indices: tail length, tail DNA%, and olive tail distance. The comet test was performed according to Singh et al.’s study [18,19].

The lymphocytes were collected immediately after 6 h of incubation and processed as follows: first, a clean frosted glass slide was placed on the horizontal operating table and preheated to 40°C, 0.6% agarose was melted and preheated to 45°C, and 100 μL of 0.6% normal melting point agarose was added to the slide. Then, the cover slide was immediately added, and it was cooled at 4°C for 10 min to solidify the gel. The cover glass was removed, 80 μL of 1% normal melting point agarose containing 20 μL of cells (mixed with a 20 μL cell suspension and 80 μL low-melting-point agarose) was added, the cover glass was immediately added, and it was cooled for 10 min at 4°C to solidify the gel. Then, the cover glass was removed, 80 μL of 1% low-melting-point agarose was added, the cover slide was added immediately, and cooled at 4°C for 10 min to make the glue solidify cover glass removed. Afterward, the slide was placed horizontally into a freshly prepared 4°C lysis buffer for 30 min. After the sliding glass was removed, the lysate was sucked dry with absorbent paper and placed on the anode end of the horizontal electrophoresis tank and precooled freshly configured electrophoresis buffer was added to the electrophoresis tank. Electrophoresis was conducted for 40 min (25 V, 300 mA). The voltage and current can be adjusted by changing the height of the electrophoresis solution. After electrophoresis, the slide was placed on a small porcelain square plate, a neutralizing solution was slowly added, and the slide was then submerged and allowed to stand for 15 min. A 50 mL syringe was used to absorb the liquid in the pan, and absorbent paper was then used to absorb the liquid on the back of the glass slide. Finally, 50 μL of ethidium bromide was added to each slide, and a cover glass was added for staining for 20 min.

A fluorescence microscope was used to randomly select 50 cells from each slide (excluding the edge of the slide) at a magnification of 200×. DNA damage was explained by analyzing the olive tail distance, tail length, and tail DNA percentage (tail DNA%) on the IMI image analysis system. All steps were performed in the dark to prevent additional DNA damage, and the slides were analyzed within 4 h.

#### 2.5.2 Micronucleus test

The micronucleus test is used for the rapid detection of chromosome abnormalities. In this test, the chromosomes of interphase cells are damaged to cause chromatids or chromosomes to break and form fragments or rings without centromeres to form one or more micronuclei outside the main nucleus in the cytoplasm at the end of the division. Among them, binuclear is one of the micronuclei. Therefore, according to the micronucleus rate under a light microscope, the severity of DNA damage caused by toxic substances can be determined. The greater the micronucleus rate, the more serious the DNA damage caused by toxic substances. The micronucleus test was performed according to Fenech et al.’s study [20]. Lymphocytes were harvested immediately after 72 h of culture and treated as follows.

The culture medium was transferred to a 10 mL centrifuge tube and centrifuged at 1,000 rpm for 7 min. The supernatant was discarded, and 0.075 mmol/L KCl (5 mL) preheated at 37°C was added and mixed well. The liquid was placed in a water bath at 37°C for 30 min, fixed with newly prepared methanol and glacial acetic acid (3:1), and a stationary solution (0.5 mL), and centrifuged at 1,000 rpm for 7 min. The supernatants were then discarded, approximately 1 mL of the hypotonic solution was left to mix with the cells, and the solution was drawn into an eyedropper. The new, fixed, 5 mL solution was added to the centrifuge tube, and the cell suspension in the eyedropper was gently injected into the fixed solution, fully mixed, and fixed three times (the first time for 30 min; the second time, it was placed in the refrigerator at 4°C overnight; and after the third centrifugation, the supernatant was removed, 0.2 mL of new stationary solution was added, and then was dropped tablets by ice water method). The 2–4% Giemsa staining solution was prepared with a buffer of pH 7.0. It was dyed for 15–20 min, rinsed with steamed water, and observed under a light microscope after drying. Fifty lymphocytes were observed in each film, and the percentages of micronuclei and binuclear cells were calculated.

#### 2.5.3 Chromosome aberration test

Chromosome aberrations represent significant genetic damage. Generally, lymphocytes in the peripheral blood will not divide under normal conditions. However, by adding an appropriate amount of PHA to the culture, the cells can enter the proliferation cycle, and a large number of mitotic cells can be obtained at this time. After the cells were treated with colchicine, the metaphase chromosome division phase was obtained. When chemically harmful substances act on cells, they can cause damage to the spindle. As a result, cell division cannot be completed, abnormal aneuploid cells are formed, and the aneuploid rate of cells has a good linear relationship with dose (concentration). When chemically harmful substances act on cells, the spindle can be damaged, resulting in the formation of abnormal aneuploid cells by the failure of cell division, and there is a good linear relationship between the aneuploid rate and the dose (concentration). Therefore, the higher the cell aneuploid rate, the more serious the chromosome damage caused by harmful substances.

The cells were harvested immediately after culturing for 72 h. Approximately 2–4 h before the cells were harvested, colchicine was added to the culture medium at a final concentration of 0.3 μg/mL. After harvest, the cells were washed twice with phosphate buffer. An appropriate amount of pre-warmed 0.075 M KCl solution was added, and hypotonic treatment was performed. The fixative (methanol–glacial acetic acid 3:1) was added for fixation, and the tablet was dropped to prepare the specimen. The slides were stained with Giemsa and dried naturally, and well-dispersed cells in metaphase were selected for observation under an oil microscope at a magnification of 1,000×. For each dose group, 50 metaphase cells were analyzed. The percentage of aneuploid peripheral blood lymphocytes was calculated.

### 2.6 Statistical analysis

The database was established in Excel 2003, and statistical analysis was performed using the SPSS 25.0 software package. The data are expressed as mean ± SE. Differences between the mean of data were assessed using one-way ANOVA and were considered significant at *P* < 0.05.

## 3 Results

### 3.1 Comet test

**Figure 2:**
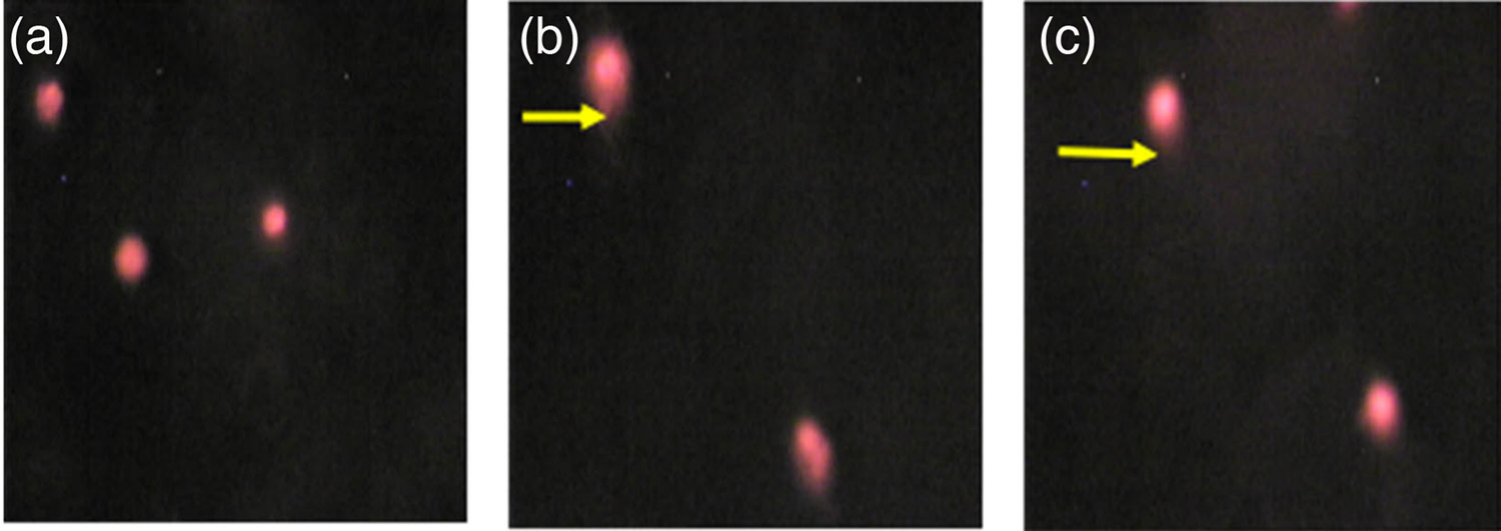
Comet test image of human peripheral blood lymphocytes treated with CdS QDs ((a) control group; (b) CdSⅠ group; (c) CdSⅡ group, 1× 200).

**Figure 3:**
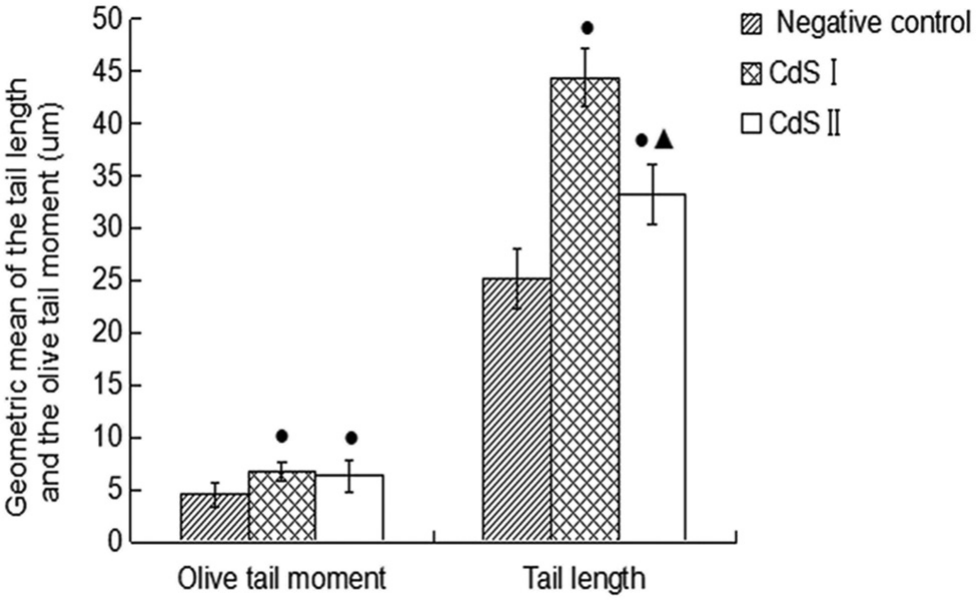
DNA damage (average ± SEM) expressed as olive tail moment and tail length. Capital letters represent statistical differences between treatments (*P* < 0.05). Note: ●: compared with the control group, *P* < 0.05; Note: ▲: compared with the *con* *P* < 0.05.

**Figure 4:**
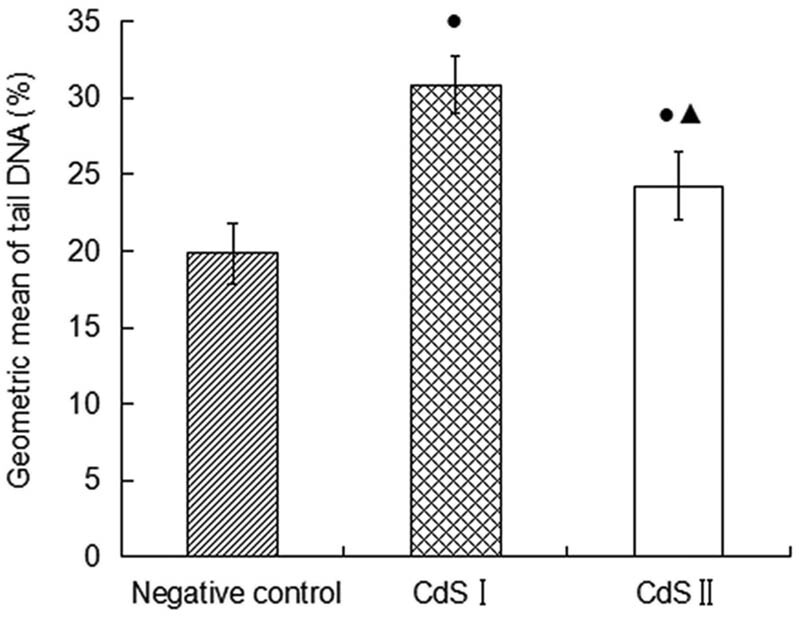
DNA damage (average ± SEM) expressed as tail DNA%. Capital letters represent statistical differences between treatments (*P* < 0.05). Note: ●: compared with the control group, *P* < 0.05; ▲: compared with the low group, *P* < 0.05.

For the comet test, the results after 6 h of exposure are shown in Figure 2. There was no tailing phenomenon in the peripheral blood lymphocytes of the control group (Figure 2a), indicating that there was no DNA damage. The peripheral blood lymphocytes of the CdS I and CdS II groups exhibited a tailing phenomenon (Figure 2b and c), indicating DNA damage. The data (Figures 3 and 4) showed that the micronucleus and dual-nucleus rates of human peripheral blood lymphocytes in the CdS I and CdS II groups were significantly different from those in the control group (*P* < 0.05). The olive tail distance, tail length, and tail DNA% of the CdS I and CdS II groups were larger than those of the control group. The olive tail distance of the CdS I group was compared with that of the CdS II group, and the difference was not significant (*P* > 0.05). The tail length and tail DNA% of the CdS I group were significantly larger than those of the CdS II group (*P* < 0.05).

### 3.2 Micronucleus test

**Figure 5:**
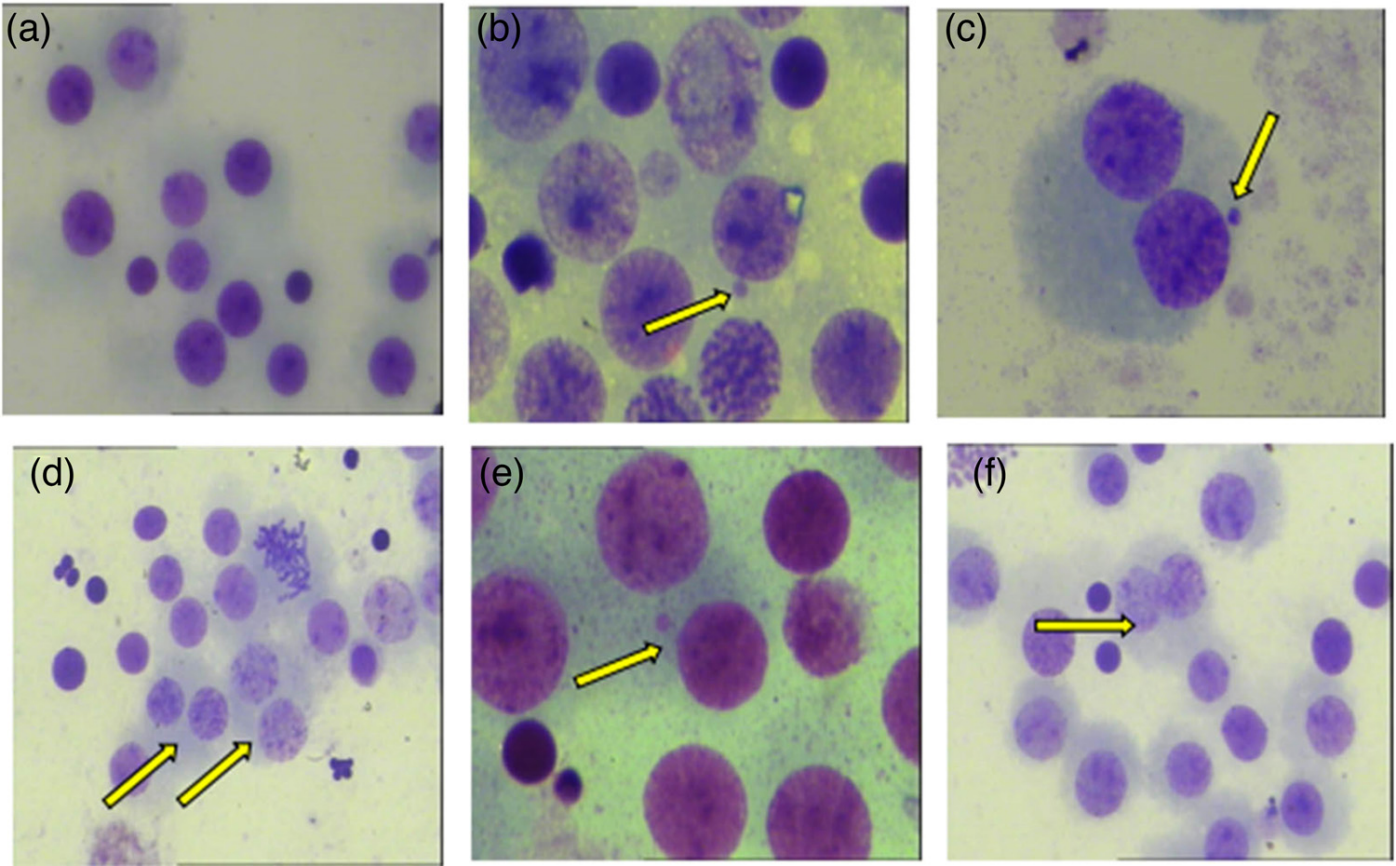
Image of micronucleus test of human peripheral blood lymphocytes treated with CdS QDs ((a): control group, ×400; (b) CdSI group, micronucleus in cells, ×1,000; (c) CdSI group, micronucleus in binuclear cells, ×1,000; (d) CdSI group, 2 binuclear cells, ×400; (e) CdS; group, micronucleus appeared in the cell, ×1,000; (f) CdS0 group, dual nucleus appeared in the cell, ×400).

**Figure 6:**
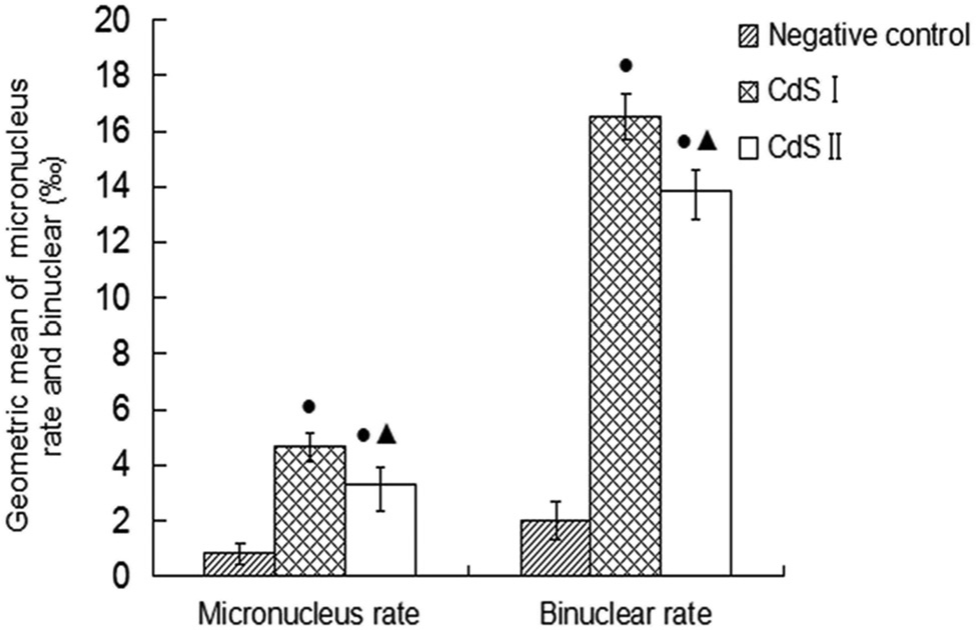
DNA damage (average ± SEM) expressed as micronucleus rate and binuclear rate. Capital letters represent statistical differences between treatments (*P* < 0.05). Note: ●: compared with the control group, *P* < 0.05; ▲: compared with the low group, *P* < 0.05.

In the micronucleus test, after 72 h of exposure, the results of each group of cells are shown in Figure 5. The peripheral blood lymphocytes of the control group were normal (Figure 5a). Micronuclei (Figure 5b and e) and dual nuclei (Figure 5c, d and f) were found in both the CdS I and CdS II groups. The data (Figure 6) showed that the micronucleus rate and dual-nucleus rate of human peripheral blood lymphocytes in the CdS I and CdS II groups were significantly different from those of the control group (*P* < 0.05). The micronucleus and dual-nucleus rates of the CdS I and CdS II groups were higher than those of the control group. The micronucleus and dual-nucleus rates of human peripheral blood lymphocytes in the CdS I group were significantly higher than those in the CdS II group (*P* < 0.05).

### 3.3 Chromosome aberration test

**Figure 7:**
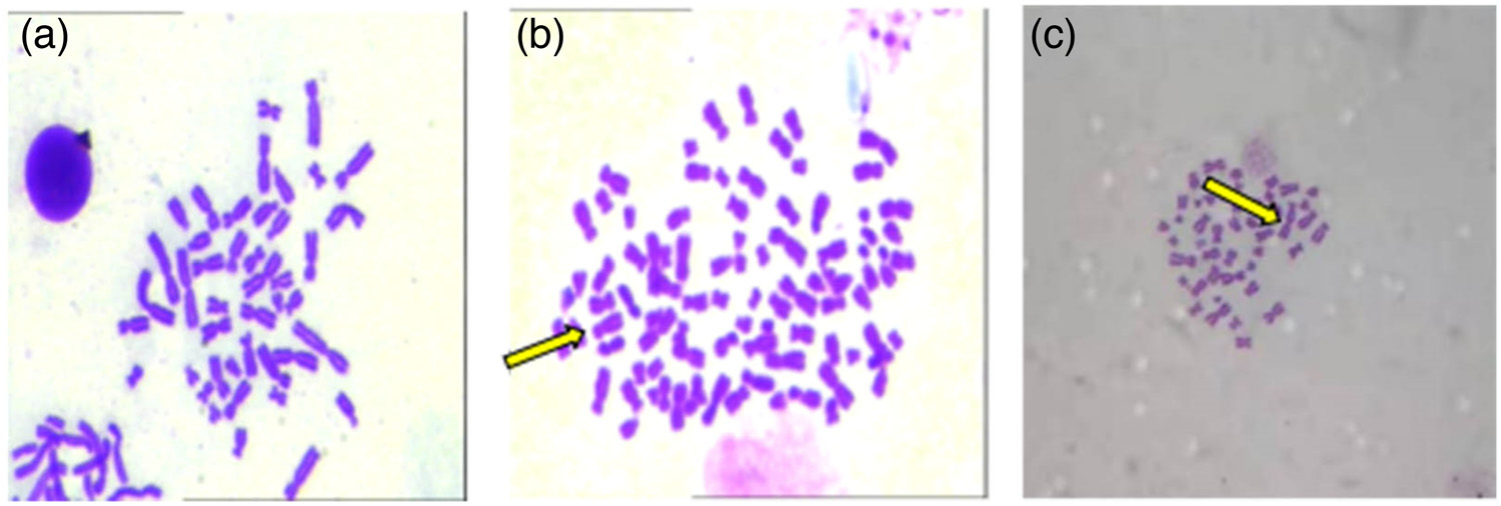
Image of chromosome aberration test of human peripheral blood lymphocytes treated with CdS QDs ((a) control group, ×1,000; (b) CdS I group, aneuploid in cells, ×1,000; (c) CdS I group, dicentric chromosomes appear in the cell).

**Figure 8:**
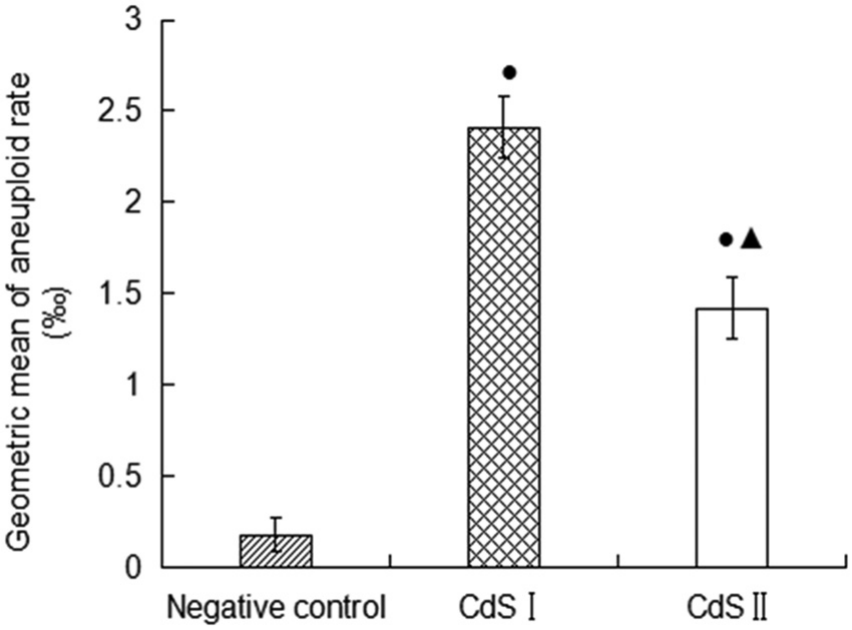
DNA damage (average ± SEM) expressed as aneuploid rate. Capital letters represent statistical differences between treatments (*P* < 0.05). Note: ●: compared with the control group, *P* < 0.05; ▲: compared with the low group, *P* < 0.05.

For the chromosome aberration test, the results after 72 h of exposure are shown in Figure 7. No aneuploids appeared in the peripheral blood lymphocytes of the control group (Figure 7a), and aneuploid and dicentric chromosomes appeared in the peripheral blood lymphocytes of the CdS I group (Figure 7b and c). Figure 8 shows that the chromosome aneuploid rate of human peripheral blood lymphocytes in the CdS I and CdS II groups was significantly higher than that in the control group (*P* < 0.05). The chromosome aneuploid rate in the CdS I group was significantly different from that of the CdS II group *(P* < 0.05), and the chromosome aneuploid rate in the CdS I group was higher than that in the CdS II group.

## 4 Discussion

CdS QDs, as nanomaterials, have attracted increasing attention as platforms, such as sensors and fluorescent probes [1]. Given the growing demand and use for CdS QDs, it is necessary to understand their potential toxicity to organisms and the environment [9]. However, there have been few reports on the genetic toxicity of CdS QDs. Therefore, it is of great importance to study the genetic toxicity of CdS QDs. In this study, CdS QDs were used as the research object. Through comet, micronucleus, and chromosome aberration tests, the damaging effect of CdS QDs on the DNA of human peripheral blood lymphocytes was comprehensively evaluated from the three aspects of single-cell DNA breakage and chromosome integrity and abnormal chromosome separation.

The DNA damage resulting from CdS QDs was evaluated by the comet assay. The results showed that the olive tail distance, tail length, and tail DNA% of the CdS I and CdS II groups were significantly larger than those of the control group, and the tail length and tail DNA% of the CdS I group were significantly larger than those of the CdS II group. This result showed that CdS QDs have a certain damaging effect on the genetic material of human peripheral blood lymphocytes, and the damaging effect after wrapping is lighter than that without wrapping. Katubi et al. [21] studied the effect of exposure dose and time of CdTe QDs on the cytotoxicity and genotoxicity of human liver cancer cells and found that with an increase in the exposure dose of CdTe QDs and prolongation of exposure time, olive tail moment and tail DNA% both significantly increased, indicating that CdTe QDs can induce DNA strand breaks. The related mechanism may be that when QDs infect lymphocytes and are absorbed by lymphocytes through endocytosis, they interact with cells to directly destroy the cellular antioxidant system, increase ROS, cause cellular oxidative stress, produce too many free radicals, attack polyunsaturated fatty acids (PUFAs) in the cell membrane, cause lipid peroxidation, and, finally, cause DNA strand breakage [22]. In addition, free radicals caused by QDs can damage DNA integrity through double-stranded nicks [23].

The ability of CdS QDs to induce chromosome breakage was evaluated by the micronucleus test. The results showed that the olive tail distance, tail length, and tail DNA% of the CdS I and CdS II groups were significantly larger than those of the control group, and the tail length and tail DNA% of the CdS I group were significantly larger than those of the CdS II group. This result shows that CdS QDs have a certain damaging effect on the genetic material of human peripheral blood lymphocytes, and the damaging effect after wrapping is less than that without wrapping. Manshian [24] showed in a study of the genotoxic capacity of CdSe/ZnS QDs with different surface chemistries that Cd/Se QDs can significantly increase the cell micronucleus rate. Demir et al. [25] used *in vitro* tests to evaluate the cytotoxicity and genotoxicity of cadmium oxide NPs. The results showed that CdO NPs could induce chromosome and DNA single- or double-strand breaks and mutations. These studies have shown that QDs can cause the formation of micronuclei. The mechanism of micronuclei formation might be that QDs damage cell chromosomes by inhibiting, slowing down, or even terminating the normal division of lymphocytes. The mechanism may also be that QDs can induce apoptosis by inducing caspase family expression, downregulating p53 and Bcl-2 gene expression, enabling the apoptosis factor Fas system, and intervening in the mitochondria-mediated apoptosis signaling pathway [26,27]. The important characteristics of apoptosis are chromatin fragmentation and chromatin pyknosis, resulting in a micronucleus formation.

The ability of CdS QDs to induce genetic damage was evaluated by a chromosome aberration test. The results showed that CdS QDs could significantly increase the aneuploid rate of cells, and the aneuploid rate of CdS QDs encapsulated by thioglycolic acid was significantly lower than that of the nonencapsulated group. George [28] studied the genotoxicity and interference of gold NPs in common *in vitro* mutagenicity and genotoxicity tests and found that gold NPs can cause chromosome aberrations. The mechanism of chromosome aberration formation may be that QDs directly destroy the antioxidant system to increase oxygen-free radicals. Excessive oxygen-free radicals can damage the spindle in the metaphase of cell division such that chromosomes cannot be effectively pulled to the two cell poles and finally induce the formation of polyploidy [29,30].

All three tests showed that the thioglycolic acid-coated CdS QDs caused significantly less damage to genetic material than the uncoated group. Other studies have shown that compared with bare QDs, QDs with a coating as a protective factor are beneficial for reducing cytotoxicity [31]. The reason may be that the coating layer can control dissolution and cellular uptake, increase the stability of QDs, and slow down their oxidation process in cells [24,32].

In summary, CdS QDs can enter human peripheral blood lymphocytes through endocytosis and exert genotoxicity. The damage to the genetic material of CdS QDs encapsulated by thioglycolic acid is significantly reduced. Therefore, QD surface modification may be an effective method to delay the harmful effects of QDs; however, the subcellular localization of QDs, low pH environment, oxidation caused by ultraviolet light penetration of skin and/or an inflammatory reaction may degrade the coating *in vivo*, and the stability and intrinsic toxicity of nanomaterials together represent the key determinants of genotoxicity induction [24]. Therefore, further research is needed to fully understand the mechanism of toxicity of CdS QDs and estimate their long-term impact on human health. Because there were few toxicity indices selected in this study, there was still a lack of molecular-level results, thus warranting further research in future studies.
